# Effect of *Pisolithus tinctorious* on Physiological and Hormonal Traits in *Cistus* Plants to Water Deficit: Relationships among Water Status, Photosynthetic Activity and Plant Quality

**DOI:** 10.3390/plants10050976

**Published:** 2021-05-13

**Authors:** Beatriz Lorente, Inés Zugasti, María Jesús Sánchez-Blanco, Emilio Nicolás, María Fernanda Ortuño

**Affiliations:** Centro de Edafología y Biología Aplicada del Segura (CEBAS-CSIC), Department of Irrigation, P.O. Box 164, 30100 Espinardo-Murcia, Spain; blorente@cebas.csic.es (B.L.); zugastilopez.i@gmail.com (I.Z.); quechu@cebas.csic.es (M.J.S.-B.); emilio@cebas.csic.es (E.N.)

**Keywords:** ectomycorrhizae, mechanisms of resistance, ornamental plants, phytohormones, water relations, water stress

## Abstract

*Cistus* species can form ectomycorrhizae and arbuscular mycorrhizal fungus that can bring benefits when plants are under water stress conditions. However, the application of some ectomycorrhizae on the water uptake under drought through physiological traits and hormonal regulation is less known. The experiment was performed during three months in a growth chamber with *Cistus albidus* plants in which the combined effect of the ectomycorrhiza *Pisolithus tinctorious* inoculation and two irrigation treatments (control and water-stressed plants) were applied. Irrigation absence caused significant decrease in aerial growth and tended to decrease soil water potential at the root surface, leading to a decrease in leaf water potential. Under these conditions, the abscisic acid and salicylic acid content increased while the precursor of ethylene decreased. Although the mycorrhization percentages were not high, the inoculation of *P. tinctorious* improved the water status and slightly cushioned the rise in leaf temperature of water-stressed plants. The ectomycorrhiza decreased the scopoletin values in leaves of plants subjected to deficit irrigation, indicating that inoculated plants had been able to synthesize defense mechanisms. Therefore, *Pisolithus tinctorious* alleviated some of the harmful effects of water scarcity in *Cistus* plants, being its use a sustainable option in gardening or restoration projects.

## 1. Introduction

*Cistus albidus* L. is a typical shrub of the Mediterranean climate. They are widely cultivated both for reforestation and for its use in Mediterranean gardening, especially in Xeriscape, where water management for saving this resource is one of the best options. These plants began to be widely used in gardening and landscaping due to their rusticity and attractive blooms.

*Cistus albidus* responds to water deficit by developing avoidance mechanisms for the regulation of transpiration based on stomatal closure, reduction of the leaf area and epinastia [1]. Furthermore, tolerance to water stress can be explained by other functional and structural adaptations of plants, such as osmotic adjustment, changes in the elasticity of the cell wall, and mineral and hormonal balance [2].

It is widely known that mycorrhizae confer physiological characteristics to plants that help them improve their water and nutritional status, especially in adverse conditions such as water stress. However, the response of these plants will depend on both the species of the plant and the type of fungus with which it is inoculated, as well as the extension, duration, and type of stress [3,4].

*Cistus* species can form both ectomycorrhizae (ECM) and arbuscular mycorrhizal fungus (AMF); they are dualistic plants [5,6,7]. The dual mycorrhization state is also present in other species such as *Populus*, *Salix*, *Eucalyptus* [5,6]. In an earlier experiment we studied whether an AMF, *Glomus iranicum* var. *Tenuihypharum*, despite being a genus (*Cistus*) that generally forms ectomycorrhizae, could bring benefits, especially when plants are under water stress conditions. In that experiment, mycorrhizae helped improve the water status of the plant when irrigation was removed, as demonstrated by the less negative leaf water potential values; it also minimized the decrease in chlorophyll levels, showing plants with higher ornamental value and increased in quantum yield (Y(II)) and photochemical quenching (qP) were related to the greater photosynthetic efficiency recorded [8].

Several studies have shown beneficial effects of ectomycorrhizal symbioses on the performance of tree species under drought stress [9]. For example, seedlings of *Picea* colonized by ECM fungi exhibited increased stomatal conductance and photosynthesis, shoot water potential and growth compared to non-mycorrhizal plants [10]. These ameliorative effects have, at least partly, been ascribed to the increasing in the absorbing surface area and the exploration of larger soil volumes by the extramatrical mycelia, as well as to the role of aquaporins which function is related to post-translational regulation and to the coordination of the phytohormones [11,12]. Transport of water and nutrient uptake through hyphae to host plants improves plant water and nutrient supply improving the performance of plants during periods of drought stress [9]. Furthermore, drought-stressed ECM-colonized plants show increased hydraulic conductance compared with non-mycorrhizal plants [13,14]. Concretely, *Pisolithus tinctorius* is one of the most widespread ECM fungi and can establish associations with a broad variety of species and has been recorded in a wide range of habitats [15]. *P. tinctorius* has great mycorrhizal capacity and provides the plant with greater efficiency in the absorption of water and nutrients [16], so it has been used in reforestation program sand and in commercial ECM inoculum production [17].

In general, mycorrhizal fungi plays an important role in regulating phytohormones such as jasmonic acid (JA), salicylic acid (SA), and ethylene (ET), which are involved in the host defense response, and abscisic acid (ABA), gibberellin (GA), and citokinin (CK), which modulate the growth of the plants. However, while the role of hormones in AMF-inoculated plants is widely known [18,19], less is known in ECM-inoculated plants, although, fundamentally, studies of the effect of ABA and SA in ECM-inoculated plants under adverse conditions have been carry out. For example, Rincón et al. [20] evaluated whether two ectomycorrhizal fungi (*Laccaria bicolorthe* and *Cenococcum geophilum*) affected ABA production in larch during osmotic stress and Luo et al. [21] determined the phytohormone changes in plants inoculated with *Paxillus involutus* under salinity. However, in the case of *P. tinctorius*, the relationships between water status and hormonal production in plants subjected to water stress is less known.

For all the above, in this study we examine how the interaction of *Cistus albidus* with the ectomycorrhizal fungus *Pisolithus tinctorious* affects water uptake under drought through physiological traits and hormonal regulation. The objectives of the study were (i) to study physiological and hormonal response to water deficit; (ii) to evaluate the effect of *P. tinctorious* on physiological traits and hormonal regulation of *Cistus* plants; and (iii) to analyze the changes in the relationships among physiological, morphological, and ornamental parameters under water deficit and mycorrhizal inoculation. Comparative data relating these physiological processes to water stress may prove beneficial toward understanding the drought tolerance of plants.

## 2. Results

### 2.1. Mycorrhization Percentage and Growth

At the end of the experiment, mycorrhizal inoculation produced a colonization of around 30% in root systems of well-irrigated *Cistus* plants. The level of colonization in roots of mycorrhizal plants decreased near to 20% with the suppression of the irrigation (Table 1). In both cases, the mycorrhization percentages were not high.

Water stress caused significant decrease in aerial growth parameters such as height and leaf DW, being the latter the one that decreases the most (30% respect to C) (Table 2). The inoculation of *Pisolithus tinctorious* increased almost all growth parameters for both irrigation conditions, as observed when we compare CM with C and WSM with WS. Indeed, inoculated plants of both irrigation treatments (CM and WSM) were taller and their leaf and root DW were higher than those corresponding to non-inoculated plants (C and WS) (Table 2).

### 2.2. Leaf Mineral Ontent

Water stress had a significant effect on leaf mineral content decreasing almost the entire mineral measured, when comparing C and WS, having a highly significant effect in Ca, Na, Mg, and Cu. The effect of the mycorrhiza treatment was hardly significant. Under well-watered conditions, only the leaf content of Ca and S in CM plants increased their values both in around 30% respect C plants (Table 3). Under water stress conditions, ECM produced a slight tendency to decrease almost all the elements of WSM plants respect to WS (Table 3).

### 2.3. Water Relations

**Figure 1:**
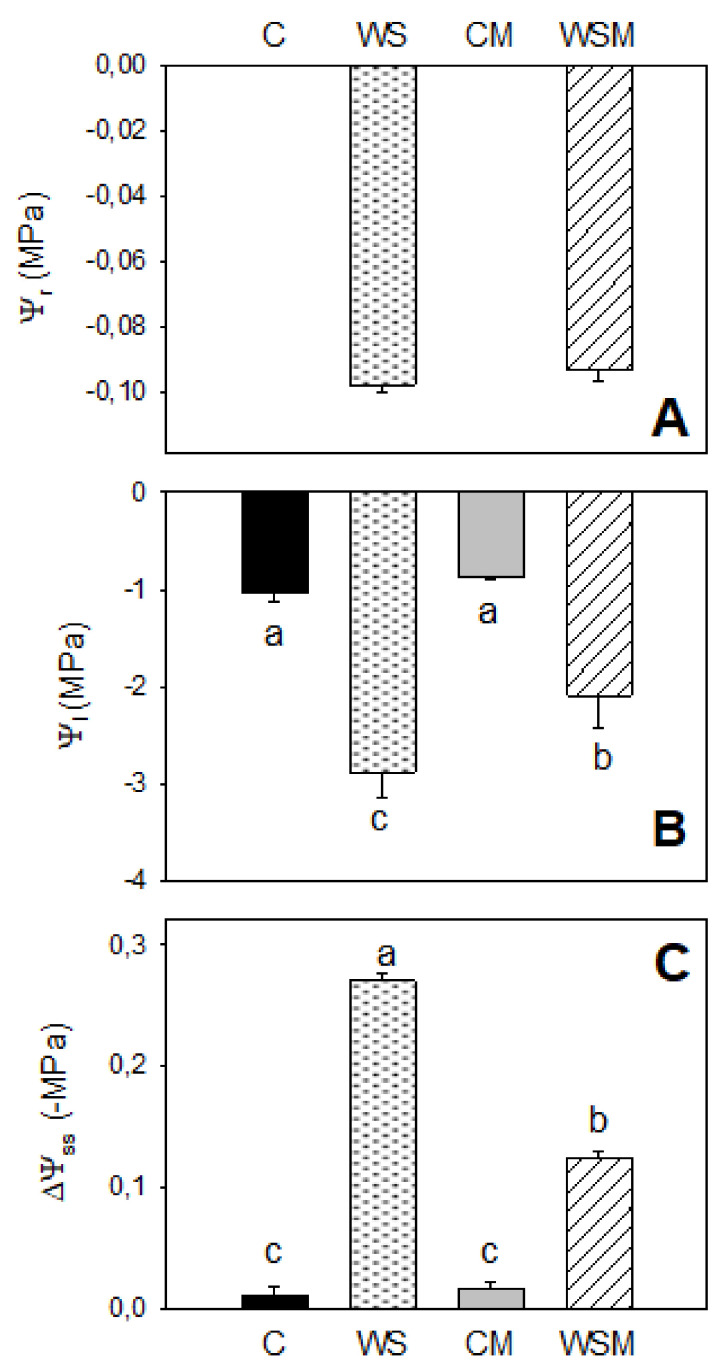
Soil water potential at the root surface (Ψ_r_) (**A**), leaf water potential (Ψ_l_) (**B**) and total seasonal leaf osmotic water potential changes due to dehydration (ΔΨ_SS_) (**C**) of *Cistus albidus* plants under well-irrigated and non-irrigated conditions, with and without *Pisolithus tinctorious* inoculation, at the end of the experiment. Values are means of three replications. The vertical bars indicate standard errors. Different lowercase letters indicate significant differences between treatments according to Duncan_0.05_ test. Absence of letter s in rows indicates no significant difference between treatments. C, well-watered plants; WS, non-irrigated plants; CM, well-watered and inoculated plants; WSM, non-irrigated and inoculated plants.

Soil water potential at the root surface (Ψ_r_) tended to decrease in WS plants due to water stress, causing greater resistance to water absorption, which leads to a decrease in leaf water potential. No differences were observed due to the effect of mycorrhizae (Figure 1A).

In well-watered plants, both inoculated and non-inoculated (CM and C, respectively), the values of leaf water potential (Ψ_l_) remained at −1.0 MPa, showing a good water status of the plants (Figure 1B). When stress was applied, non-inoculated plants (WS) decreased Ψ_l_ significantly, reaching minimum values of −3.0 MPa. The use of *Pisolithus tinctorius* alleviated significantly the negative effect of water stress and the fall in values was not so severe (−2.1 MPa) (Figure 1B).

Figure 1C shows the contribution to dehydration to change in osmotic water potential (ΔΨ_SS_). Under well-watered conditions, ΔΨ_SS_ was almost negligible for both inoculated and non-inoculated plants (C and CM, respectively). Water stress caused a decrease in ΔΨ_SS_, reaching minimum values of around −0.27 MPa in non-inoculated plants (WS), while the ECM dampened this drop (−0.12 MPa).

### 2.4. Gas Exchange

**Figure 2:**
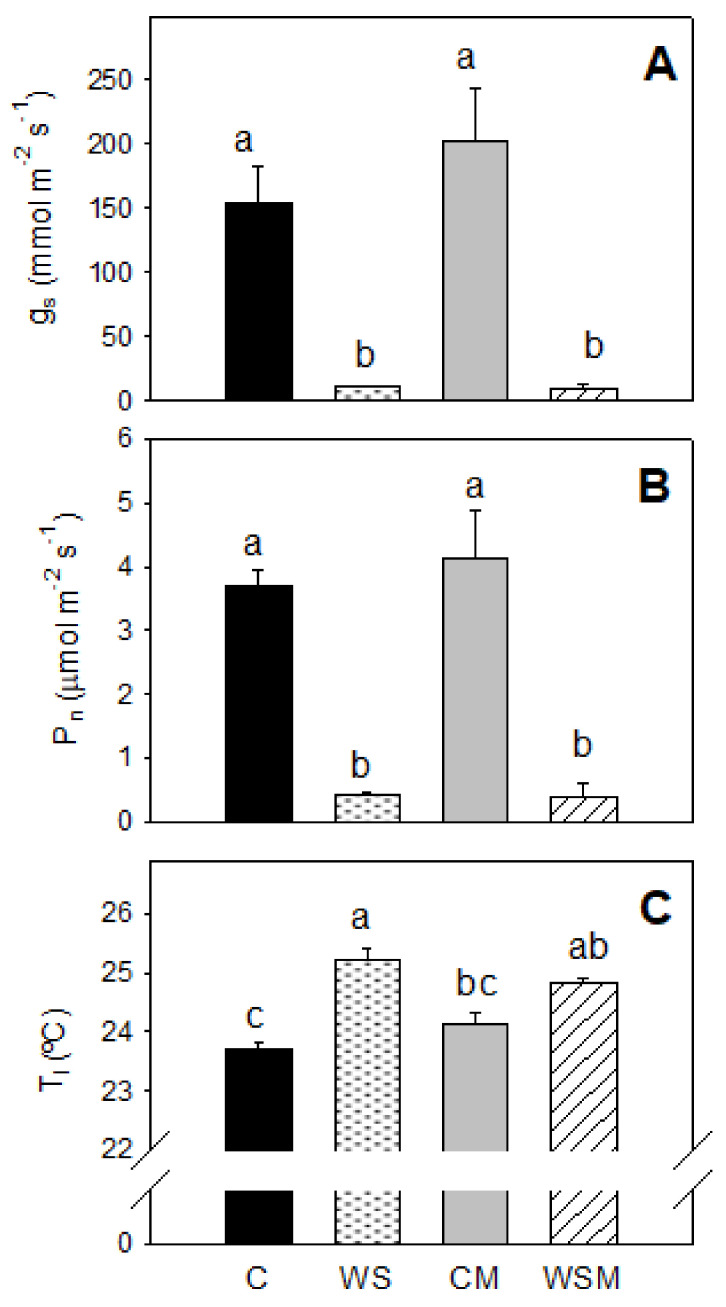
Stomatal conductance (g_s_) (**A**), net photosynthetic rate (P_n_) (**B**) and leaf temperature (T_l_) (**C**) of *Cistus albidus* plants under well-irrigated and non-irrigated conditions, with and without *Pisolithus tinctorious* inoculation, at the end of the experiment. Values are means of three replications. The vertical bars indicate standard errors. Different lowercase letters indicate significant differences between treatments according to Duncan_0.05_ test. C, well-watered plants; WS, non-irrigated plants; CM, well-watered and inoculated plants; WSM, non-irrigated and inoculated plants.

Under water stress conditions, the stomatal conductance (g_s_) fell drastically, almost reaching the stomatal closure in WS plants. Differences of 150 mmol m^−2^ s^−1^ between C and WS treatments were observed (Figure 2A). *Pisolithus tinctorious* did not favor opening in either case, neither under conditions of good irrigation nor under water deficit (CM and WSM). Net photosynthesis (P_n_) showed a behavior very similar to that of g_s_ (Figure 2B). Leaf temperature (T*_l_*) of plants subjected to water stress (WS) was 1.5 °C higher than C plants (Figure 2C).

### 2.5. Phytohormones

**Figure 3:**
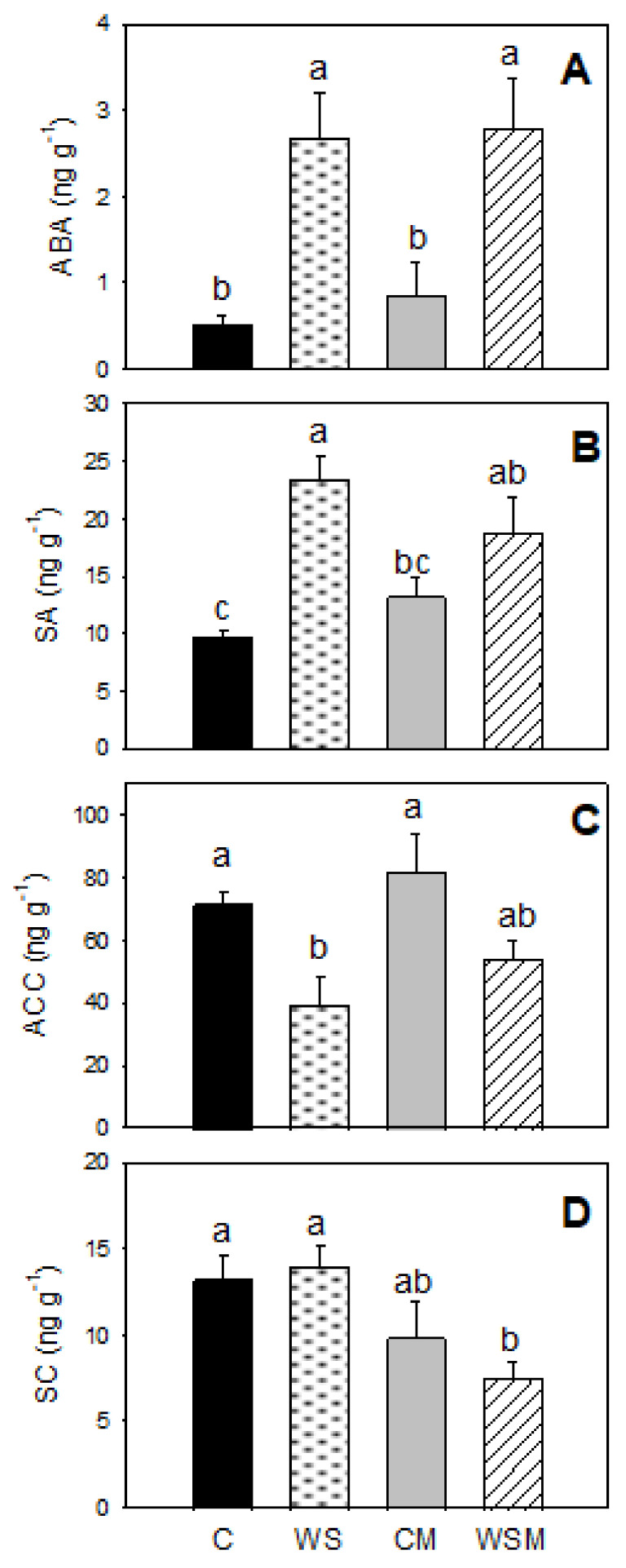
Abscisic acid (ABA) (**A**), salicylic acid (SA) (**B**), precursor of ethylene (ACC) (**C**), and scopoletin (SC) (**D**) of *Cistus albidus* plants under well-irrigated and non-irrigated conditions, with and without *Pisolithus tinctorious* inoculation, at the end of the experiment. Values are means of three replications. Different lowercase letters indicate significant differences between treatments according to Duncan_0.05_ test. The vertical bars indicate standard errors. C, well-watered plants; WS, non-irrigated plants; CM, well-watered and inoculated plants; WSM, non-irrigated and inoculated plants.

At the end of the experiment period, the abscisic acid (ABA) and salicylic acid (SA) content in WS leaves were approximately 5 and 3 times as high as those in control leaves, respectively (Figure 3A,B). By contrast, the precursor of ethylene (ACC) content was decreased significantly by the irrigation suppression (45% of C) (Figure 3C). Mycorrhizae only had a significant effect on scopoletin (SC) under water stress conditions (WSM), significantly decreasing their values by approximately 46% respect to non-inoculated plants (WS) (Figure 3D).

### 2.6. Relationships between Physiological, Morphological and Ornamental Parameters

**Figure 4:**
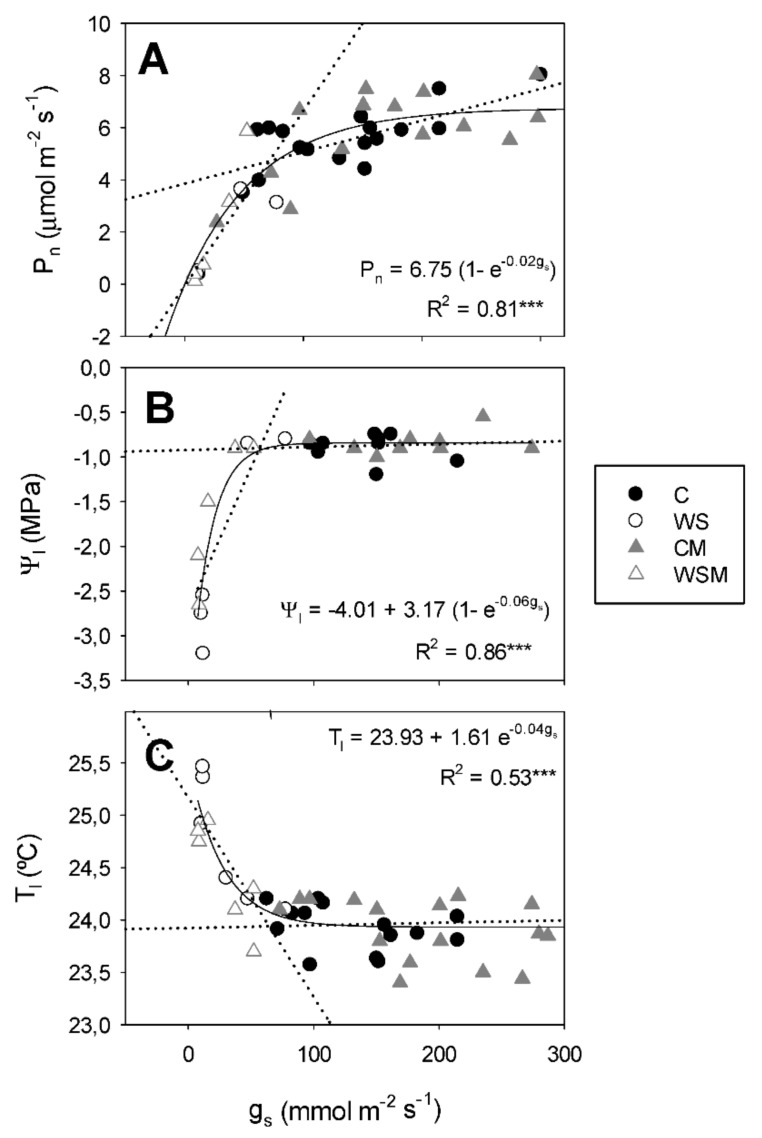
Solid lines represent the relationships between stomatal conductance (g_s_) and net photosynthesis (P_n_) (**A**), g_s_ and leaf water potential (Ψ_l_) (**B**) and g_s_ and leaf temperature (T_l_) (**C**) in *Cistus albidus* plants under well-irrigated and non-irrigated conditions, with and without *Pisolithus tinctorious* inoculation, during the experimental period. Dotted lines represent the linear regressions of the control (C and CM) and water stress treatments (WS and WSM) individually. Values are means of three replications. C, well-watered plants (closed circles); WS, non-irrigated plants (open circles); CM, well-watered and inoculated plants (closed triangles); WSM, non-irrigated and inoculated plants (open triangles). *** significant at *p* < 0.001.

The relationship between g_s_ and P_n_ was well represented by an exponential function when all the values of the four treatments obtained during the experimental period were considered (Figure 4A). Highly significant linear regressions between g_s_ versus P_n_ can be obtained when considering control treatments (C and CM) and water stress treatments (WS and WSM) individually, suggesting that g_s_ explain differences in P_n_ within each irrigation treatment. A strong correlation (R^2^ = 0.86) between g_s_ and leaf water potential (Ψ_l_) was observed, fitting with an exponential sigmoid model. Ψ_l_ of plants subjected to water stress (WS and WSM) declined sharply (variation between −1.0 and −3.2 MPa) in a narrow range of g_s_ (Figure 4B). Relationship between T_l_ and g_s_ fits with an exponential decay model. Leaf temperature was maintained between 23.3 and 24.3 °C when the stomatal conductance exceeded approximately 65 mmol m^−2^ s^−1^ (Figure 4C).

With increasing water stress, the dependence of *Ψ_l_* and T*_l_* on g*_s_* increased, as well as the dependence of the relative chlorophyll content (RCC) on P*_n_*. WS as compared to C promoted intrinsic water use efficiency (WUE, P_n_/g_s_). However, P_n_ was less dependent on shoot dry weight when the plants were subjected to irrigation suppression (Table 4). Under water stress conditions, *Pisolithus tinctorious* only had effect on the relation between RCC and P_n_, being WSM higher than WS (Table 4).

## 3. Discussion

The percentage of mycorrhization of *Pisolithus tinctorius* was not excessively high, despite being an ectomycorrhiza, which has special relevance in this genus with high mycorrhizal capacity [6]. In our conditions, the cistus seedlings were cultivated in pots, which prevented the extension of the fungal mycelium and of the ECM roots. Imposed water stress meant a reduction in the percentage of mycorrhization, like plants, fungi are also affected by water limitation [22]. Studies of dually colonized plant species such as members of the genus *Cistus* indicate that ECM may be more sensitive to drought than AMF [23,24]. In fact, in a previous study with AMF (*Glomus iranicum* var. *tenuihypharum*) in *Cistus albidus* plants under water stress [8], the percentage of mycorrhization was 65%, more than double that obtained with *Pisolithus tinctorius*.

Drought is one of the main adverse factors for seasonal plants, especially for plants grown in pots [1,25,26]. At the end of the experiment, water stress caused a decrease in leaf dry weight, which could be interpreted as a conservative strategy to reduce transpiration and maintain hydraulic conductivity under water depletion [27,28,29]. Despite the low percentage of mycorrhization, ECM improved both shoot and root growth of the plants under well-irrigated conditions. Furthermore, unlike what occurred in *Cistus* plants inoculated with AMF [8], *Pisolithus tinctorious* increased root growth under water stress. Usually, more root branching has been found in ECM species than AMF, what is related to higher ability to absorb more water and nutrient, improving plant water relations under low water conditions [30,31,32,33,34].

Leaf water potential and actual osmotic potential are good indicators of water stress [35,36]. In our study, plants subjected to water stress showed no osmotic adjustment, perhaps, the speed of the development of water stress and the low inorganic solutes accumulation, such as potassium and sodium, could not contribute to osmo-regulation [8,37]. The fact that ΔΨ_SS_ at the end of the experiment in control treatment were almost null indicated that dehydration was the major mechanism involved in ΔΨ_SS_, while the more noteworthy ΔΨ_SS_ in WS treatment seems to indicate that the dehydration could be produced by the transpiration [38]. Soil water potential at the root surface (Ψ_r_) reflects the accumulation of net solutes allowing reduction of Ψ_l_ (around −3.0 MPa), in order to guarantee water transport to the leaves [37,39,40,41]. Concerning ECM effect, it improved the plant water status under stress conditions [42]. This ameliorative effect has been ascribed to the extended external mycelia of the root systems of ECM fungi, which reach soil pores inaccessible to the roots in water [5,43,44].

As a consequence of the reduction of the substrate water potential at the root surface and the leaf water potential in plants subjected to water stress, the stomatal conductance decreased drastically, acting as a mechanism to prevent excessive loss of water [25,26,45,46,47]. In our conditions, the relationship between g_s_ and Ψ_l_ was adjusted to an exponential curve, so that under water-stress plants exhibited progressively lower Ψ_l_, but almost no change in g_s_. Likewise, it has been well established that plants regulate rates of transpiration and photosynthesis in parallel, maintaining a balance between g_s_ and P_n_ [48]. A strong correlation between stomatal conductance with net photosynthesis observed in the current study appears to reflect the gas exchange limitation of photosynthesis [49,50,51]. At the end of the experiment, the imposed water stress induced an increase in intrinsic WUE, which is in agreement with Costa et al. [46], who reported that deficit irrigation strategies can be successfully applied to different crops, in order to improve water savings. Water deficit by closing stomata, causes increasing leaf temperature, considering the major determinant of leaf temperature is the rate of evaporation or transpiration from the leaf [52,53,54]. This behavior was observed at the end of our experiment.

ABA synthesis is one of the fastest responses of plants to abiotic stress causing stomatal closure. In addition, SA is involved in the regulation of drought responses [55], inducing the generation of reactive oxygen species (ROS) and, consequently, causing the stomatal closure [56]. In our experiment, endogenous ABA and SA levels in stressed plants increased up to of six and almost twofold than those of control plants, respectively, while endogenous 1-aminocyclopropane1-carboxylic acid (ACC) levels in leaves decreased. ACC is the precursor for the production of ethylene and under drought, a close relationship between ACC expression and ethylene synthesis has been demonstrated [57]. In these conditions, ACC levels decrease and, therefore, plant growth is reduced. However, some studies suggest that ABA and ethylene are antagonists and have described decreases in ethylene production as a consequence of an increased concentration of ABA in water stressed plants, regulating some drought responses in plants, such as root and leaf growth [58,59,60,61,62]. This is in accordance with our results, which show that in conjunction with a decrease in stomatal conductance and leaf water potential, ABA in leaves increased, while ethylene decreased, increasing root growth.

Hormonal profiles are also altered to alleviate the negative effects of water stress on mycorrhizated plants [32,63,64,65]. Several studies confirmed that inoculation with mycorrhizal fungi results in decreasing the endogenous ABA and SA [20,66]. However, in our assay ECM treatments (CM and WSM) had not significant changes in any of both leaf hormones neither under well-watered nor water stress conditions. Since ABA is known to induce stomatal closure [67], its lower concentration in ECM plants could play a role in the stomatal conductance, allowing no increase in net CO_2_ assimilation rate in ECM inoculated plants [17]. This fact leads no significant improvement in intrinsic water use efficiency (P_n_/g_s_).

Nevertheless, ECM significantly decreased leaf scopoletin (SC). Scopoletin (6-methoxy-7-hydroxycoumarin) is a typical phytoalexin which is an important secondary metabolite synthesized in plants as a defense mechanism against various environmental stresses [68]. Its synthesis is activated once some kind of infection has occurred in plants, but it can also be triggered due to various types of abiotic stresses. Scopoletin has also been found in many other plant species (e.g., Solanaceae, such as tobacco or potato, and sunflower, among others), showing antifungal and antibacterial activity [68,69,70]. Its accumulation has been correlated with resistance to microbial attack and other stresses, as well as mechanical damage and dehydration [71]. Furthermore, it appears to be the product that most increases in concentration in infected plants compared to other coumarins and coumaric glycosides, such as scopolin, esculetin, and esculin [72,73,74]. In our study, SC decreased significantly in mychorrhizal plants indicating that ECM plants have been able to synthesize defense mechanisms against abiotic and biotic stresses against non-mycorrhizal plants, and therefore SC is lower in ECM plants.

In conclusion, the mechanism of *C. albidus* to avoid water stress was related to its ability to decrease aerial growth and to modify leaf gas exchange, increasing water use efficiency. Water stress positively stimulated the levels ABA and SA, who strongly enhanced drought tolerance. On the other hand, despite the low percentages of mycorrhization, *Pisolithus tinctorious* inoculation in addition to improving plant growth, and slightly gas exchange and quality plant parameters, it improved the water status of *Cistus* under water stress conditions, probably due to a decrease in SC values, suggesting that the selected ECM can alleviate some of the harmful effects of water scarcity. Therefore, the use of *Cistus* plants inoculated with *Pisolithus tinctorious* in gardening or restoration projects can be a sustainable economic and environmental option.

## 4. Materials and Methods

### 4.1. Plant Material and Experimental Conditions

Forty-eight plants of *Cistus albidus* L. from the nursery were used. On January 18, 2017, these plants were transplanted into 1.5 L pots with a commercial substrate based on Sphagnum peat, coconut fiber and perlite in an 8:7:1 ratio and were placed in a controlled growth chamber. The climatic conditions of the chamber were those necessary for optimal growth: temperature (23 °C/18 °C, day/night), photosynthetic photon flux density (350 μmol m^−2^ s^−1^), photoperiod (16h/8h, light/dark), and relative humidity (RH) (60%). Plants were watered at field capacity.

### 4.2. Treatments

After two weeks of acclimatization, half of the plants (24) were inoculated with the ectomycorrhizal fungus *Pisolithus tinctorius*. Two months later, once the plants were stabilized, the irrigation of half of inoculated and non-inoculated plants were removed for a month, while the remaining plants continued to be irrigated at field capacity. Thus, the following treatments were established: C, Control, well-watered plants; CM, well-watered and inoculated plants; WS, plants subjected to withholding; WSM, inoculated plants subjected to water stress. Each treatment included three replications. Each replication consisted of four plants. The experiment lasted three months.

### 4.3. Fungal Colonization

At the end of the experiment, roots samples with the surrounding rhizosphere soil were collected to treat to evaluate fungal development. Three root samples were used in each replication. The percentage of mycorrhizal root colonization was estimated as following: Once cleaned, the roots were immersed in KOH (100 °C for 7 min), followed by a bath in H_2_O_2_ (100 °C, 5–6 min) and finally, trypan blue staining (4 min). The percentage of colonization was calculated using the methodology proposed by Kormanik and McGraw [75]. The colored roots were placed on specialized plates for counting and were observed under the magnifying glass by counting 100 fields neatly (positive and negative). The percentage of colonization was calculated using the following formula proposed by Sieverding [76]:

%colonization = (number of colonized fields/total number of fields observed) × 100(1)

### 4.4. Leaf Mineral Content

At the end of the experimental period, the inorganic mineral content of dry leaves was determined in three plants per replication by means of emission spectrophotometry. The nutrient concentrations were determined in a digestion extract with HNO3:HClO4 (2:1, *v/v*) by Inductively Coupled Plasma optical emission spectrometer (ICP-OES IRIS INTREPID II XDL, Thermo Fisher Scientific Inc., Loughborough, UK).

### 4.5. Biomass and Height

At the end of the experiment, three plants per replication were selected and all the substrate was gently washed from their roots. After washing the substrate from the roots, the plants were individually separated into leaves, stems and roots and the fresh weight of each organ was determined. After that, each sample was dried in an oven at 80 °C, until samples reached a constant weight, and the dry weights (DW) were obtained.

Every 15 days, the height of three plants per replication was measured. Plant height was measured from the base of the plant at the substrate surface to the most distal growth.

### 4.6. Water Relations

Leaf water potential (Ψ_l_), leaf osmotic potential (Ψ_s_) and osmotic water potential at full turgor (Ψ_100s_) were determined in three plants per replication throughout the experimental period. The leaf water potential was determined during light hours according to the technique described by Scholander et al. [77], using a pressure chamber (Model 3000; Soil Moisture Equipment Co., Santa Barbara, CA, USA), the leaves were pressurized at a rate of 0.03 MPa s^-1^. Ψ_s_ and Ψ_100s_ was measured using a Wescor 5520 vapor pressure osmometer (Wescor Inc., Logan, UT, USA), according to Gucci et al. (1991) [78]. For Ψ_100s_ the leaf samples were previously subjected to a rehydration treatment by dipping their petioles in distilled water for 24 h to achieve complete saturation.

Changes in Ψ_s_ (ΔΨ_s_) and in Ψ_100S_ (ΔΨ_100S_) were calculated according to Girma and Krieg [79] as the difference in Ψ_s_ and Ψ_100S_ measured at the initial (i) and at the end (e) of water stress period.

ΔΨ_S_ = (Ψ_s_)^i^ − (Ψ_s_)^e^ (2)

ΔΨ_100S_ = (Ψ_100S_)^i^ − (Ψ_100S_)^e^ (3)

The contribution of dehydration to changes of Ψ_S_ (ΔΨ_SS_) was calculated using the following equation:

ΔΨ_SS_ = ΔΨ_S_ − ΔΨ_100S_ (4)

Soil water potential at the soil-root interface (Ψ_r_) was computed according to Jones (1983) [80]:

Ψ_r_ = (Ψ_l_^WS^ − Ψ_l_^C^) × g_s_^WS^/g_s_^C^ (5)

where, Ψ_l_^WS^ and Ψ_l_^C^ correspond to the mean value of the leaf water potential in the WS and C treatments, respectively, and the g_s_^WS^ and g_s_^C^ correspond to the mean value of the respective treatments. A value of Ψ_r_ was calculated for each of the three drought pots using the value of Ψ_l_^C^ and g_s_^C^. The Ψ_r_ is assumed to be zero for control plants.

### 4.7. Gas Exchange and Thermography

Stomatal conductance (g_s_) and net photosynthesis rate (P_n_) were measured with the LI-COR 6400 portable meter (LI-COR Inc., Lincoln, NE, USA). The flow rate of circulating air within the system was approximately 200 mmol s^−1^, with a leaf vapour pressure deficit to air of about 2 KPa. The CO_2_ concentration was fixed at 380 ppm and the photosynthetically active radiation (PAR) at 600 µmol m^−2^ s^−1^. The measurements were carried out in three plants per replication on the same days as the water relations.

At the same time and in the same plants that gas exchange was determined, leaf temperature (T_l_) was measured with an infrared camera (FLIR-e50 System, Inc., Danderyd, Sweden) which consisted of a 240 × 180 pixels line scan imager operating in the 7.5e13 mm region, with a noise equivalent temperature difference of 0.05 °C at 30 °C and an accuracy of 2 °C or 2% of the reading. The background temperature, distance of the camera from the canopy, air temperature, emissivity, and relative humidity were used as input at the start of each series of measurements; so, the camera automatically corrects for atmospheric transmission based on these data. Background temperature was determined as the temperature of a crumpled sheet of aluminium foil in a similar position to the leaves of interest with the emissivity set at 1.0. Emissivity for leaf measurements was set at 0.96 [81] and the distance at which images were taken was 0.5 m. The images were processed with ThermaCam Researcher Professional 2.10 software (FLIR Quick Report, Danderyd, Sweden).

### 4.8. Relative Chlorophyll Content

The relative chlorophyll content (RCC) was determined periodically in three plants per replication with a Minolta SPAD-502 chlorophyll meter (Konica Minolta Sensing Inc., Osaka, Japan), a non-destructive method.

### 4.9. Hormonal Determination

The extraction and analysis of the plant hormones were developed in agreement with Albacete et al. [82] including slight modifications in the protocol, such as the measurement of scopoletin (phytoalexin). Firstly, 100 mg of fresh plant material (leaf) was homogenized in liquid nitrogen and placed in 0.5 mL of cold (−20 °C) extraction mixture of methanol/water (80/20, *v*/*v*). The mix was centrifuged at 15,000 r.p.m. (20,627× *g*), 4 °C for 15 min. The supernatants were kept at 4 °C and the remaining plant material (pellet) was re-extracted with additional 0.5 mL of the same extraction solution. The mix was re-centrifuged at 15,000 r.p.m. (20,627× *g*), 4 °C for 15 min. Pooled supernatants were passed through a Chromafix C_18_ cartridge (previously activated with 3 mL methanol/water (80/20, *v*/*v*) to remove interfering lipids and plant pigments and evaporated to dryness (3 h, approximately). The residue was dissolved in 200 µL metanol/water (80/20, *v*/*v*), then sonicated during 8 min and filtered through Millex filters with 0.45 µm pore size nylon membrane (Millipore, Bedford, MA, USA). The final sample extracted was injected into an ultra-high-performance liquid chromatography (UHPLC) coupled triple quadrupole mass spectrometry (UHPLC-QqQ-MS/MS).

The separation of plant hormones and phytoalexins was developed with slight modifications in accordance with Albacete et al. [82] by using UHPLC coupled to a 6460 triple quadrupole mass spectrometer (Agilent Technologies, Waldbronn, Germany), and a BEH C_18_ column (2.1 × 50 mm, 1.7 µm) (Waters, Milford, MA, USA) with a guard column (2.1 × 5.0 mm, 1.7 µm). The column temperature was 40 °C. Water/acetic acid (99.99:0.01, *v:v*) (solvent A) and acetonitrile (solvent B) were used as mobile phases at the flow rate of 0.2 mL min^−1^. The injection volume was 10 µL. The gradient program used was: 19% B at 0 min, 90% B at 2.5 min, 90% B at 4.5 min, 19.0% B at 6 min, and 19% B at 8.0 min for column equilibration. The electrospray interface (ESI) was set up in the negative and positive mode and the mass spectrometry analysis were run in the multiple reaction monitoring modes (MRM). The ionization and fragmentation conditions were as follows: gas temperature 325 °C, gas flow 8 L/min, nebulizer 45 psi, sheath gas temperature 375 °C, jet stream gas flow 11 L/min, capillary voltage 4000 V and 2750 V (positive and negative mode, respectively), and nozzle voltage 1000 V and 1500 V (positive and negative mode, respectively) according to the most abundant product-ions. The quantitative evaluation of plant hormones and scopoletin was carried out using authentic standards. The standards used are included in Table 5 and were diluted in water/methanol (20:80, *v*/*v*) for the quantification curves.

### 4.10. Statistical Analysis

In the experiment, 48 plants were randomly attributed to each treatment, with three replications for each treatment. The data were analysed by one-way ANOVA using Statistical Package for the Social Sciences (IBM SPSS Statistics 26 for Windows, CA, USA). Treatment means were separated with Duncan’s Multiple Range Test (*p* ≤ 0.05). Prior to the statistical analysis, percentage of root colonization was subjected to an arcsine square-root transformation to ensure the homogeneity of the variance. Relationships between parameters were fitted to different regressions using SigmaPlot v. 14.5 software (SPSS Inc., Chicago, IL, USA).

## Figures and Tables

**Table 1:** Percentage of root colonization in plants subjected to control and water stress with *Pisolithus tinctorious* (CM and WSM, respectively) at the end of the experiment. Values are means of three root samples per replication.

	Mycorrhization Percentage (%)
CM	29.3 a
WSM	21.05 b

Different letters indicate significant differences according Duncan _0.05_ test.

**Table 2:** Dry weight of leaf, stem, root and shoot, root/shoot ratio, and height of *Cistus albidus* plants under well-irrigated and non-irrigated conditions, with and without *Pisolithus tinctorious* mycorrhiza, at the end of the experiment. Values are means ± SEM (*n* = 3).

	TREATMENTS			
	C	WS	CM	WSM
Leaf DW (g)	3.32 ± 0.39 b	2.37 ± 0.31 c	5.59 ± 1.44 a	4.06 ± 0.41 ab
Stem DW (g)	2.25 ± 0.30	2.05 ± 0.22	3.58 ± 1.05	2.80 ± 0.15
Root DW (g)	1.64 ± 0.15 c	1.55 ± 0.20 c	2.05 ± 0.13 b	3.17 ± 0.30 a
Shoot DW (g)	5.58 ± 0.68 bc	4.43 ± 0.54 c	8.50 ± 0.89 a	6.85 ± 0.55 ab
Root/shoot ratio	0.30 ± 0.03 b	0.35 ± 0.01 ab	0.22 ± 0.03 b	0.48 ± 0.09 a
Height (cm)	33.42 ± 1.35 b	30.50 ± 1.08 c	35.83 ± 1.46 a	33.23 ± 1.49 b

DW, dry weight; C, well-watered plants; WS, non-irrigated plants; CM, well-watered and inocu-lated plants; WSM, non-irrigated and inoculated plants. Different letters in rows indicate significant differences between treatments according Duncan_0.05_ test. Absence of letters in rows indicates no significant difference between treatments.

**Table 3:** Leaf mineral content of *Cistus albidus* plants under well irrigated and non-irrigated conditions, with and without *Pisolithus tinctorious* mycorrhiza, at the end of the experiment. Values are means ± SEM (*n* = 3).

	TREATMENTS			
	C	WS	CM	WSM
	Macronutrients			
Ca (%)	0.86 ± 0.02 b	0.60 ± 0.03 c	1.00 ± 0.05 a	0.49 ± 0.05 c
K (%)	3.19 ± 0.23 a	2.72 ± 0.50 ab	3.02 ± 0.32 a	2.18 ± 1.28 b
P (%)	0.67 ± 0.06 ab	0.59 ± 0.03 ab	0.73 ± 0.06 a	0.54 ± 0.04 b
Na (%)	0.14 ± 0.02 a	0.08 ± 0.01 b	0.16 ± 0.01 a	0.05 ± 0.01 b
S (%)	0.17 ± 0.03 b	0.16 ± 0.02 b	0.22 ± 0.01 a	0.15 ± 0.01 b
Mg (%)	0.35 ± 0.02 a	0.26 ± 0.02 b	0.37 ± 0.01 a	0.24 ± 0.01 b
	Micronutrients			
Cu (mg/Kg)	12.95 ± 1.29 a	4.97 ± 0.82 b	10.85 ± 2.23 a	4.15 ± 0.45 b
Fe (mg/Kg)	68.78 ± 7.44 ab	54.68 ± 4.96 b	82.97 ± 10.13 a	102.85±35.34 a
Mn (mg/Kg)	117.13 ± 3.41 ab	123.52 ±28.55 ab	153.05 ± 7.71 b	108.93 ± 23.75a
B (mg/Kg)	165.35 ± 3.29	158.55 ± 1.40	162.75 ± 3.98	161.10 ± 7.69
Zn (mg/Kg)	87.95 ± 13.71 a	59.58 ± 9.09 ab	72.73 ± 5.42 ab	41.97 ± 7.37 b

C, well-watered plants; WS, non-irrigated plants; CM, well-watered and inoculated plants; WSM, non-irrigated and inoculated plants. Different letters in rows indicate significant differences between treatments according Duncan_0.05_ test. Absence of letters in rows indicates no significant difference between treatments.

**Table 4:** Water indices related to gas exchange and photosynthetic efficiency at the end of the experiment. Values are means ± SEM (*n* = 3).

	TREATMENTS			
	C	WS	CM	WSM
P_n_/g_s_ (μmol CO_2_/mol H_2_O)	26.21 ± 4.41 b	38.58 ± 3.87 a	23.31 ± 8.62 b	40.55 ± 2.10 a
−Ψ_l_/g_s_ (MPa/mol H_2_O m^−2^ s^−1^)	5.40 ± 0.23 b	170.10 ± 45.75 a	4.73 ± 1.05 b	205.97 ± 33.10 a
T_l_/g_s_ (°C/mol H_2_O m^−2^ s^−1^)	166.2 ± 32.61 b	2215.92 ± 82.63 a	130.34 ± 27.72 b	2576 ± 50.02 a
RCC/P_n_ (%/μmol CO_2_ m^−2^ s^−1^)	10.10 ± 0.57 c	102.60 ± 8.24 b	10.20 ± 2.17 c	127.95 ± 11.16 a
P_n_/Shoot DW (μmol CO_2_ m^−2^ s^−1^/g)	0.63 ± 0.05 a	0.10 ± 0.01 c	0.34 ± 0.02 b	0.06 ± 0.02 c

P_n_, net photosynthesis; g_s_. stomatal conductance; Ψ_l_, leaf water potential; T_l_, leaf temperature; RCC, relative chlorophyll content; Shoot DW, shoot dry weight; C, well-watered plants; WS, non-irrigated plants; CM, well-watered and inoculated plants; WSM, non-irrigated and inoculated plants. Different letters in rows indicate significant differences according Duncan_0.05_ test.

**Table 5:** Plant hormones and phytoalexins: reference, brand, and solubility.

Hormone	Reference	Laboratory	Solubility
Jasmonic acid	14631-10 MG	SIGMA	DMSO (16 mg/mL)
trans-Zeatin	Z0876-5 MG	SIGMA	DMSO (3 mg/mL)
trans-Zeatin	001030n (5 mg)	OLCHEMLM	DMSO (3 mg/mL)
[2H5]-trans-Zeatin	030030n (1 mg)	OLCHEMLM	DMSO (3 mg/mL)
Camalexin	SML1016-5 MG	SIGMA	DMSO (20 mg/mL)
Isopentenyladenine	SC-279669 (1G)	SANTA CRUZ	DMSO (20 mg/mL)
trans-Zeatin riboside	SC-20846 (5 MG)	SANTA CRUZ	ETHANOL/DMSO (78.95:21.05, *v/v*) (50 mg/mL)
trans-Zeatin riboside	001031n (5 mg)	OLCHEMLM	DMSO
trans-Zeatin glucoside	SC-237225 (1MG)	SANTA CRUZ	ETHANOL/DMSO (75.00:25.00, *v*/*v*) (50 mg/mL)
trans-Zeatin 9-glucoside	001047n (1 mg)	OLCHEMLM	DMSO
Abscisic acid	SC-238015 (50 MG)	SANTA CRUZ	ETHANOL (50 mg/mL)
3-indoleacetic acid	SC-254494 (5 G)	SANTA CRUZ	ETHANOL (50 mg/mL)
1-aminocyclopropane-1-carboxylic acid (ACC)	SC-202393 (500 MG)	SANTA CRUZ	WATER (437 mg/mL)
Giberellic acid (GA1)	012249n (1 mg)	OLCHEMLM	ETHANOL (50 mg/mL)
Giberellic acid (GA3)	SC-257556 (500 MG)	SANTA CRUZ	ETHANOL (50 mg/mL)
Giberellic acid (GA4)	SC-235248 (5 MG)	SANTA CRUZ	ETHANOL (50 mg/mL)
Giberellic acid (GA5)	SC-490117 (25 MG)	SANTA CRUZ	ETHANOL (50 mg/mL)
Giberellic acid (GA7)	012254n (1 mg)	OLCHEMLM	ETHANOL (50 mg/mL)
Salycilic acid	SC-203374 (100 G)	SANTA CRUZ	ETHANOL (50 mg/mL)
Scopoletin	SC-206059 (50 MG)	SANTA CRUZ	DMSO (30 mg/mL)

All the phytohormones were determined in three plants per replication at the end of the experimental period.

## Data Availability

The data presented in this study are available on request from the corresponding author.
